# Perspectives and practices of personnel involved in family planning with women at reproductive risk

**DOI:** 10.1590/1980-220X-REEUSP-2023-0001en

**Published:** 2023-10-30

**Authors:** Gilberto Jasso Padrón, Yesica Yolanda Rangel Flores

**Affiliations:** 1 Universidad Cuauhtémoc, Licenciatura en Medicina, San Luis Potosí, México.; 2 Universidad Autónoma de San Luis Potosí, Facultad de Enfermería y Nutrición. Maestría en Salud Pública, San Luis Potosí, México.

**Keywords:** Family Development Planning, Reproductive Rights, Noncommunicable Diseases, Health Knowledge, Attitudes, Practice, Qualitative research, Planejamento Familiar, Direitos Sexuais e Reprodutivos, Doenças não Transmissíveis, Conhecimentos, Atitudes e Práticas em Saúde, Pesquisa Qualitativa, Planificación Familiar, Derechos Sexuales y Reproductivos, Enfermedades no Transmisibles, Conocimientos, Actitudes y Prácticas en Salud, Investigación Cualitativa

## Abstract

**Objective:**

To analyze the perspectives and practices of personnel involved in family planning with women at reproductive risk due to chronic diseases.

**Method:**

Qualitative study in which physicians and nurses from primary care centers in a state in central Mexico were interviewed. Interviews were transcribed and analyzed under the Grounded Theory proposal.

**Results:**

The perspectives and practices in family planning interventions are unilaterally framed in the biomedical model. Three categories of analysis emerged: “The battle”, “Convince by imposing”, “Monitor them and catch them”.

**Conclusion:**

It is necessary to promote competencies in interculturality, gender perspective and human rights to make the intervention more of a counseling and less of a prescription for life projects.

## RESUMEN

**Objetivo:**

Analizar las perspectivas y prácticas del personal que interviene en planificación familiar con mujeres en riesgo reproductivo por enfermedades crónicas.

**Método:**

Estudio cualitativo, se entrevistó a médicos y enfermeras de centros de primer nivel de atención en un estado en el centro de México. Las entrevistas fueron transcritas y analizadas bajo la propuesta de la Teoría Fundamentada.

**Resultados:**

Las perspectivas y prácticas en la intervención en planificación familiar se enmarcan de forma unilateral en el modelo biomédico. Emergieron tres categorías de análisis: “La batalla”, “Convencer imponiendo”, “Vigilarlas y atraparlas”.

**Conclusión:**

Es necesario impulsar competencias en materia de interculturalidad, perspectiva de género y derechos humanos, para hacer de la intervención más una consejería y menos una prescripción de proyectos de vida.

## RESUMO

**Objetivo:**

Analisar as perspectivas e práticas dos profissionais envolvidos no planejamento familiar com mulheres em risco reprodutivo por doenças crônicas.

**Método:**

Estudo qualitativo no qual foram entrevistados médicos e enfermeiros de centros de atenção primária de um estado da região central do México. As entrevistas foram transcritas e analisadas sob a proposta da Teoria Fundamentada nos Dados.

**Resultados:**

As perspectivas e práticas na intervenção em planejamento familiar se enquadram unilateralmente no modelo biomédico. Emergiram três categorias de análise: “A batalha”, “Convencer pela imposição”, “Vigiar e pegar”.

**Conclusão:**

É necessário promover competências em interculturalidade, perspectiva de gênero e direitos humanos para tornar a intervenção mais um aconselhamento e menos uma prescrição de projetos de vida.

## INTRODUCTION

Indirect maternal deaths have increased in the last decade, associated with an epidemiological transition characterized by the exponential growth of Chronic Noncommunicable Diseases (CNCDs) in young women^(1)^. In Mexico alone, arterial hypertension affects 34.3% of the population between 20 and 69 years of age^(2)^ while diabetes mellitus currently affects 12%, and is expected to reach a prevalence of 18% by 2030^(3)^.

It is the obligation of the State to protect and guarantee women’s right to reproductive autonomy, which implies not only deciding freely when and how many children to have, but also developing agency to make conscious decisions about their reproductive processes^(4)^. In this direction, women must be informed of their reproductive risk, so they can access personalized counseling with full awareness. Although reproduction is a fundamental right, in the case of women with CNCD, the physiological adaptations occurring in pregnancy, childbirth and puerperium come to represent a high-risk situation and even a probable cause of death^(5)^.

Within the aforementioned context, health personnel’s skills to assess risk, communicate it and provide reproductive counseling are fundamental for users diagnosed with some type of CNCD. The lack of skills of health personnel in the field of family planning has been documented in several studies, pointing out the violation of human, sexual and reproductive rights, and even the practice of obstetric violence. Among these studies, those offering repeated evidence of how the communication between health personnel and users is structured from positions of power that violates women users of services stand out with particular relevance^(6)^. Even with advances in policies, women continue to be made invisible, ignored, and are not given credibility, merit, or the ability to express an opinion^(7)^.

The studies on sexuality in people with CNCD are particularly relevant, mainly because a significant number of these document the bodies of women as asexual from the experience of the disease^(8)^, placing the biological aspect as the principle of the decrease in interest and sexual practice.

In the context of the aforementioned studies, little has been addressed in relation to the performance of health personnel, particularly with women of reproductive age diagnosed with some CNCD. Our study intends to contribute precisely to this knowledge gap. The objective is to analyze the perspectives and practices applied by health personnel in family planning interventions specifically with women who face reproductive risk associated with chronic diseases.

## METHOD

### Type of Study

Qualitative, framed in symbolic interactionism and the actor-centered perspective. These were identified as relevant because according to such theories, meaning is the product of the social convention glimpsed in social interaction^(9)^.

### Population

Purposive sampling was used in the selection of Health Centers. This strategy consists of considering cases which, by their characteristics or experiences, are known beforehand to have the greatest possible richness of information about the research phenomenon. The choice was to work in urban centers of primary care with the largest number of personnel, and those that more or less frequently offered training and continuing education in the field of Family Planning.

### Selection Criteria

Physicians and nurses working in the care of women of reproductive age with a chronic disease, with one or more years of experience in the area, who decided to participate voluntarily in the study. Health personnel were selected by intentional sampling^(10)^, and the number of informants was determined based on the theoretical saturation criterion. This is achieved when the information collected does not bring anything new to the “development of the properties and dimensions of the categories of analysis”^(11)^.

### Data Collection

Individual interviews were conducted between June 2021 and May 2022, based on a semi-structured interview script. They were performed by the first author in the medical offices, lasted approximately 40 minutes and no more than one meeting with interviewees was needed.

### Data Analysis

The audio-recorded interviews were transcribed and analyzed based on the grounded theory proposal^(12)^. The information was triangulated with the notes collected from the non-participant observation and the field diary.

### Ethical Aspects

The study was reviewed, approved, and monitored by the State Research Ethics Committee of the Health Services of the State of San Luis Potosí under registry number SLP/07-2019. The informed consent signature was collected, anonymity was guaranteed and respected, and participants were also informed about their freedom to participate or stop doing so at the time they deemed necessary. For the purposes of this publication, names have been omitted and only a number was assigned to participants.

## RESULTS

Twelve professionals were interviewed; eight women and four men, five with a degree in medicine and seven in nursing. They mentioned that women diagnosed with hypertension, diabetes, hypothyroidism, etc. who visit their health center “rarely” get pregnant, and family planning issues are not addressed in follow-up consultations for the disease. On the other hand, they acknowledge that chronicity-related issues are not assessed in gynecological consultations either.

*These patients have their consultation in the basic nucleus because of the chronic problem, the gynecologist does not assess chronic problems, and here we do not assess those of the gynecologist... these women are more in the nucleus* [where the exclusive treatment of the chronic condition is performed]. (Participant 8)

*These women already have another profile, the priority is to control the disease, avoid complications, they have other follow-up needs, planning is not a priority.* (Participant 11)

Statements such as those above are made even when they know of cases of pregnancy in users with chronic diseases.

*There is a 14-year-old patient, she is in the last trimester of her pregnancy and she is an insulin-dependent diabetic, she has type I diabetes... she does not come here for pregnancy control and she visits the central* [hospital] *for disease control.* (Participant 3).

*Very young women, adolescents, and apart from being obese, it implies a greater risk of starting a pregnancy with diabetes, or starting with preeclampsia.* (Participant 12)

In addition, they showed a tendency to limit the risk based on age groups, insisting on classifying adolescents separately from adult women, devaluing the risk in the latter by assuming that they are “of reproductive age”, and making invisible the risk situation implied by the chronic disease condition itself.

In the same way, their tendency to a greater recognition of the risk linked to lifestyles, as in women who abuse substances and/or experience situations of intrafamily violence. Regarding comorbidities, diseases that compromise mental health are characterized as the “most complicated”.

*When you know they consume alcohol, or worse, some kind of drug, the pregnancy obviously is not going to end well, or worse, it is not going to end at all.* (Participant 9)

*Mentally ill patients! and sometimes the problem is the family members who believe that they do not require [a contraceptive method], but we have had pregnant women and not necessarily because of a rape but rather they have given their consent and become pregnant, giving them a method is the problem.* (Participant 10)

Regarding the implementation of family planning, three categories of analysis were identified, which we titled “The battle”, “Convince by imposing”, “Watch them and catch them”.

## The Battle

The family planning intervention is equated with a battle and carries a *negative meaning* within the context in which it occurs. It is referred to as a situation that results from the lack of compliance with the prescription made by the health personnel, hence, when the prescription is followed, expressions such as “*yes, it is possible*”, “*it is achievable*” emerge, with a *positive meaning* within the context of family planning, and its negative counterpart “it is lost (the battle)”, which is even related to the “*loss of patients*” or leads to “*high-risk pregnancies*”. Proof of this is found in the following speech fragments.

*It is a battle that we have to continue until the end, sometimes we succeed. Sometimes they arrive after the pregnancy, and* [we say] *What happened? What are you going to choose? Which method? – “It’s just that I haven’t thought about it yet”,* [then] *we insist again.* (Participant 4)

*It is a battle, they do not understand the complications that can happen with the pregnancy, in each consultation we repeat the same thing.* (Participant 9)

## Convince by Imposing

Intervening in family planning implies getting into the work of *convincing* and *raising awareness*, that is, of “*finding the way*” or the “*manner*” to make the contraceptive method accepted, which almost always entails “*insisting*”. The strategies are diverse, screening when women visit other departments or while they are in line to vaccinate their children, telephone follow-up or implementing workshops on contraceptive methods inside and outside the health center.

“*Letting oneself be convinced*” would seem to be related to “*being aware*”, but rather, it is related to “*making them aware*”, that is, an action seen as positive, which in turn is related to “*being a good patient*” since now she “*knows about the risks*” of not accepting a method.

*The most difficult thing is to convince the patient, you give her an explanation, you tell her the reason, the reason why she must change certain issues in her life, and that is a challenge, what you really have to do is convince that if she does it, there will be a positive change in her life.* (Participant 8)

*We make them see the risk (...) I’ve had people come and say “I want to get pregnant” - Do you want to get pregnant?* (asks in a discouraging manner, making a disapproving gesture), *How old are you? “no more than 38-40” -ah ok- Are you diabetic or hypertensive? “Not that diabetic”, she manages with us, very well -and then what is going to happen? Do you want to have a baby that also has diabetes, or do you want to get sick and stay that way? I had a case of a lady that would insist she wanted to get pregnant and got pregnant, yes, it is very difficult, you will have to, it is very difficult sometimes to make patients see it.* (Participant 5)

*Convincing* implies deploying strategies that health personnel use to reach an end, the use of some method or avoiding pregnancy. As previously described, approval is equivalent to users being “*aware*”. The act of implementing family planning is described as an action of *raising awareness*.

*Sometimes the goals are not met, but we insist a lot, a lot, you have no idea how much we insist, but sometimes there are people who say “no, I’m not going to plan”, and well, after a while I see them again pregnant here.* (Participant 4)

## Watch them and Catch them

*Surveillance* also implies a series of follow-up strategies seeking to guarantee users’ attendance at the health center. The use of means such as cell phones (the context in the neighborhoods where they work is perceived as violent) and home visits stand out; the health center is necessarily flexible, extensible and extra mural.

*We talk to patients who are absent, we tell them, your method has arrived, because sometimes we spend like two or three months without injectable hormones, so when they arrive, we talk to them directly.* (Participant 3)

*If you miss your appointment, we have a strategy, you know. What happened? Why didn’t you come to your consultation? “Well, no, because it was raining, because I couldn’t, or I went to my girl’s kindergarten...” We schedule another appointment, we don’t leave them, just like, she didn’t come anymore, well, no way. No, we schedule their appointment or we talk by phone, normally we perform home visits, but it is an area that is no good for doing home visits, so it is better to use the phone.* (Participant 1)

Watching also implies questioning, as a Family Planning strategy, women of reproductive age who simply pass by somewhere in the Center. They are made “*captives*” or “*seized*” for counseling.

*We catch many adolescents, we also catch the high-risk ones, almost like that, more or less, I hold almost all of them captive after an obstetric event, they are the ones I see the most, regardless of whether they are minors or adults, all of them.* (Participant 12)

Making them captive clearly comes from a war analogy, reward for battles. In this case, the meaning of these actions is given a different name, they are resignified for operational aspects in health services, although closely related to the already described *battle* and *watching*.

## DISCUSSION

The perspectives are framed almost unilaterally in the biomedical model. By focusing exclusively on the biological aspect, this paradigm annuls the psychosocial dimensions, making invisible the cultural traits giving particularity to existence. In turn, this leads to a standardized and depersonalized healthcare approach, implementing actions in the form of institutional mandates that are more like control and imposition practices than counseling^(13)^.

The staff invests a large part of their time and energy seeking to incorporate users into the family planning program, in an experience narrated as exhausting, confrontational, and unsatisfactory, but above all, worrisome. It is described as a situation of conflict with the evident lack of tools for intercultural negotiation.

From a biomedical-centered perspective, it is logical to identify an ambivalent professional position towards reproductive risk. At the same time that they recognize it and even exaggerate it in their professional imaginary, they fail to bring it to reality when consulting women.

This inconsistency between imagination and practice has also been reported by other authors, who allude to the fact that although there is talk of reproductive risk in women with chronic diseases, at the time of their consultations this does not make a difference, neither means a specific way of acting. These women are usually approached with the same interest and arguments as those adopted with the rest of women^(14,15)^.

We also identified the health personnel’s tendency to emphasize the risk in the specific case of adolescents. Although this population group faces a higher risk due to their inconclusive biopsychosocial maturation, according to findings from previous studies, they do not play a leading role in the reproductive risk-CNCD relationship, since most women with reproductive risk associated with CNCD were concentrated among age groups of 30, 40 years and over^(16,17)^.

Another population group identified as predominant in the discourse on reproductive risk and CNCD is that of women with mental illnesses, in whom planning is seen more as a control strategy than a human rights strategy. This makes invisible the vulnerability faced by this group of women in relation to sexual abuse and violence, and such a situation has been previously reported by other authors^(18)^.

In addition, the fragmentation of healthcare deriving fundamentally from the biomedical perspective was documented. The hegemonic medical model fragments the body to understand the mechanisms of the disease, and believes the same can be done with how the processes of health, disease and care are experienced. Although fragmentation can be a useful strategy to understand physiological and pathological mechanisms, and can enhance depth and specialization in knowledge, it is definitely not a strategy that favors the holistic and comprehensive perspective sustaining the sexual and reproductive experience. Not only it fails to make it feasible, but this vision also divides, fragments and limits the understanding of the social phenomena framing the experience of health, illness and care, generating emotions of frustration and incompetence in health personnel^(19)^.

We realized there is no reflective approach to family planning. Consequently, the intervention does not flow in the form of counseling, but more according to the prescription and installation of methods, aiming to prescribe sexuality and the reproductive project, and annulling its dimension of being a fundamental human right. Reproduction cannot be prescribed as it is done with diseases, it is imperative to recognize contraception as one of the fundamental human rights that requires an approach including the perspectives of gender, human rights and interculturality^(20)^.

Health personnel should be aware that ensuring access to contraceptive methods in this specific group of women goes far beyond complying with institutional goals and policies. It basically implies guaranteeing reproductive health and maintaining life, providing women with accurate, scientific and timely information, so they can build their reproductive project. This consists not in giving up the maternity desire, but rather in being able to exercise their right to become pregnant and have a safe delivery with the possibility of survival and the healthy development of their children. We must renounce, as indicated by Palma^(21)^, the obsolete vision that violates human rights, in which family planning in women with reproductive risk is limited to controlling, restricting, avoiding and discouraging births.

The perspectives and dispositions motivating personnel to intervene in family planning are not in any way linked to the framework of sexual and reproductive rights. This may result from the health personnel’s vision based on the hegemonic medical model being quite limited with respect to the human-social dimension; “public health personnel do not intervene or do so to a limited extent on structural conditions (economic-political and ideological-cultural)”^(22)^.

From the previous point of view, we can understand that in spite of the search to “raise awareness” of women regarding the risks that make health personnel “battle”, the truth is that nothing will be achieved if the structural conditions maintaining this situation are not recognized and addressed, if actions continue to be guided without reflecting on the relationships maintained with users and the nature of communication established with them. In other words, if it is not triggered from being culturally competent, a tool that enables not only better results in the health counseling intervention, but also greater satisfaction and less anxiety when meeting others, which in cultural terms are significantly different^(23)^.

This cultural difference leads to the occurrence of encounters between health personnel and the user population in contexts of impositions and resistance. The explanations for this resistance are mostly framed by a positivist look and in a certain way, that reproduces the thought of hegemonic medicine. Other studies identify the existence of a series of “negative perceptions about the use”^(24)^ without reflecting on the contexts and only describing the upward trend in the use of contraceptive methods in different contexts.

In this study we observe a link between women’s resistance and a domain anchored to culture, which is incompatible with the health personnel’s perspectives of users, i.e., a domain of social stereotypes and taxonomies based on behaviors and an ethnocentrism stemming from the Hegemonic Medical Model. Therefore, it would be necessary to reflect on the role of counseling and coverage at this point, since it only starts from one perspective and not from the other, which is where it is intended to influence.

Another relevant issue that requires urgent discussion is how reproduction is seen separated from eroticism and violence in the imaginary of health personnel. It is taken for granted and based on the idea that pregnancy occurs as a result of an autonomous and individual decision, over which there is always control to decide. The existence of social pressures is not recognized, no wonder, since the *mother-wife* identity has been reported as one of the most desirable for women in many contexts. You cannot be sexually active if you do not assume the social mandate of being a mother, which can have such weight that women can practically be forced to get pregnant, even knowing that this could result in their death^(25)^.

Forced sexual encounters were not mentioned either, reinforcing the already made statement regarding the lack of perspective on gender and human rights. The need for the use of a method so that women with mental illnesses do not get pregnant is discussed, when the problem is that the society and the State do not meet the conditions to guarantee the rights of these women not being abused or raped. When seeking to legitimize the use of contraceptive methods so that women with mental illness do not get pregnant, the violence of which they are part is being legitimized.

It seems that women with CNCDs are conceived as asexual, especially women called “old” or those with a mental illness: sexuality is a topic used only for reproductive purposes and in a negative way, since there are pregnancies. Therefore, their knowledge is “restricted in relation to the sexualities of people with chronic diseases, and the health care process is inconsistent with the real needs of these human groups”^(26)^.

From a disease-focused biological perspective, it is expected that action in family planning occurs based on the disease and not on women, their desires, projects and daily lives. It is assumed that women diagnosed with CNCD are obliged to develop their life project based on the disease itself, that is, from a recognition of *being ill*, and from this, change their life project. And not the other way around, that is, by adapting the chronic disease to their life project in order to live as they want to live. Hence, women’s resistance to the prescription of reproduction and the use of contraceptive methods continues to be documented.

According to our findings on how interventions in the field of family planning work, we dare to affirm that these programs represent another of the captivities in which women live; they must accept that their bodies are monitored and prescribed. They must think about their life project from their reproductive potential and their diseases, their history is the history of their body and they live to meet the demands of others^(27)^, even others who are not there, the unborn.

The surveillance carried out by health personnel over women became evident, and although this *health surveillance* is truly not exercised exclusively over women’s health but in general over the population, “through the mediation of medicine, pedagogy and the economy, sex became not only a laic issue, but a state issue; even more: a matter in which the entire social body, and almost each one of its individuals were urged to watch themselves”^(28)^. This biopolitical vigilance clearly has a gender connotation, which is that from the hegemonic medical model, bodies are interpreted and intervened based on their social construction made based on gender; the woman’s body is hyper medicalized and with that her life project is also determined. Medicine began to monitor the body of women during menstruation, pregnancy, and menopause, and also to explain their moods based on their hormonal cycles, a condition that is not imposed over the bodies or lives of men^(29)^.

Although the perspectives and practices that support the family planning interventions seem to be a personal matter for each health actor, it is about a whole structure of thought produced and reproduced from a hegemonic medical model also supported by capitalist and neoliberal economic models according to which, in addition to being carriers of the demographic bonus, women are social actors, as well as economic.

Locating “*surveillance*”, “*battle*” or “*raising awareness*” as subcategories that make up family planning forces us to turn to decolonial studies, because the Hegemonic Medical Model emerges and is strengthened precisely from the Western thought, in which users are naturalized into a purely biological point of view and where social control measures are implemented, seeking to transform them into an “*other*” and to this end, they must be made “*captives*”.

This gives rise to a whole conception of users, who are described and classified (transformed and signified) in levels of poverty, “*low education*” and possessing a “*low level of culture*” as a way of differentiation, which deprives them from the recognition of their capacity for agency, their desires, their opinions, experiences, and of an equally valid world view.

Family planning is then configured as a colonizing strategy without being culturally competent. Family planning is imposed as colonization, it seeks to colonize the other through the impregnation of a particular world view without taking into account the world view of women and their contexts, hence the resistance imposed by women, assuming the risks (a risk not built from their world), from discrete to the most complex practices of resistance, because “where there is power, there is resistance”^(28)^.

The shared narratives make it possible to account for the need and urgency for a movement of reproductive health policies in Mexico towards an approach of human rights, interculture and gender. They must be dislodged from the colonialist approach, a situation that has begun to occur with the incorporation of the six cross-cutting axes in the current Specific Program for Sexual and Reproductive Health 2020–2024, which alludes to the importance of acting from transversal perspectives of Human Rights, Gender Equality, Interculturality, Youth, Masculinities and Population Approach^(30)^.

However, the results of this study give rise to questioning not only the performance of health personnel involved in reproductive health counseling, but also the professional skills in which health human resources are trained. Just as it is necessary to train and sensitize professional service personnel, it is also necessary to restructure training programs, integrating social and human perspectives into the curricula that make it possible to understand sexuality in its broadest complexity, questioning the hegemonic biomedical model from which the bodies and lives of women have been historically thought.

One of the most important limitations of this study is related to the obstacles to perform the participant observation in meetings between health personnel and women, a strategy we consider incorporating in future approaches, as the necessary analysis codes can be generated and help to understand the trend of social interactions between users and professionals.

## CONCLUSION

Health personnel consider family planning as an action performed to convince-impose, guide, offer, raise awareness, control, monitor and catch so that users decide the number of children they want, as well as a battle that “must be won”. Sexuality is classified based on users’ diseases and age; the first is related to “decrease” or “asexuality” and the second to a restriction, always visualized from reproduction. There is little reflection on these aspects and on family planning, which is why counseling is based on a series of assumptions and gender stereotypes from a series of classifications of users.
